# Barriers and facilitators to using standardised diagnostic assessments in child and adolescent mental health services: a qualitative process evaluation of the STADIA trial

**DOI:** 10.1007/s00787-025-02678-w

**Published:** 2025-03-18

**Authors:** Louise Thomson, Kristina Newman, Colleen Ewart, Anupam Bhardwaj, Bernadka Dubicka, Tamsin Marshall, Julia Gledhill, Alexandra Lang, Kirsty Sprange, Kapil Sayal

**Affiliations:** 1 University of Nottingham, Nottingham, UK; 2 Institute of Mental Health, Nottinghamshire Healthcare NHS Foundation Trust, Nottingham, UK; 3 Nottingham Trent University, Nottingham, UK; 4 Cambridge and Peterborough NHS Foundation Trust, Cambridge, UK; 5 University of York, York, UK; 6 Pennine Care NHS Foundation Trust, Ashton-Under-Lyne, UK; 7 Berkshire Healthcare NHS Foundation Trust, Bracknell, UK; 8 Central and North West London NHS Foundation Trust, London, UK

**Keywords:** CAMHS, DAWBA, STADIA, Process evaluation, Child and adolescent mental health, Qualitative

## Abstract

The STADIA trial aimed to assess the effectiveness of a standardised diagnostic assessment tool (Development and Wellbeing Assessment, DAWBA) in aiding clinician-made diagnosis decisions in Child and Adolescent Mental Health Services (CAMHS). This study reports the qualitative process evaluation of the STADIA trial, which aimed to identify barriers and facilitators to using the online-completed DAWBA in CAMHS. Qualitative data were collected through 109 semi-structured interviews with young people, parents/carers, healthcare professionals and service commissioners/funders in 8 CAMHS sites across England. Deductive thematic analysis was guided by the domains of the Consolidated Framework for Implementation Research. Young people and parents/carers showed high levels of engagement with the DAWBA. They perceived a validation of symptoms from the generated DAWBA report, which they actively used as ‘evidence’ when seeking help from other services. Clinicians involved in determining referral acceptance/rejection decisions were positive about its use and saw benefits in aiding decision-making. In contrast, however, barriers to clinicians engaging with the DAWBA report during the assessment stage arose from limited awareness and accessibility to the report, a context of high workload and pressure, and general concerns about the value of a diagnosis. The DAWBA was not widely used by clinicians in the expected way to aid diagnostic decision-making. However, it may offer children and young people much-needed engagement during long waiting times for initial assessment in CAMHS. The DAWBA may be more acceptable to clinical teams in triaging referrals to help with timely decisions about the most suitable services.

**Trial registration** ISRCTN15748675 (29/05/2019).

**Supplementary Information:**

The online version contains supplementary material available at 10.1007/s00787-025-02678-w.

## Introduction

The demands on Child and Adolescent Mental Health Services (CAMHS) in the UK National Health Service (NHS) exceed service capacity, leading to long waiting lists and frequent rejections of referrals into those services [1]. Long waiting list times place considerable burden on families and carers and have a negative impact on children and adolescents, for example, exacerbating mental and physical health symptoms [2] and impacting on engagement with services [3].

The use of evidence-based tools and processes to help with timely referral processing and assessments is critical in supporting effective and fast treatment. However, there are well-documented challenges to implementation of innovations in health services [4–7]. Previous studies in CAMHS have identified high levels of demand on services and limited capacity in clinical teams as key barriers to engaging clinical staff with new tools and innovations designed to improve efficiency and services [8, 9].

The current study is a nested process evaluation investigating the use of a standardised diagnostic assessment (SDA) tool (the Development and Wellbeing Assessment (DAWBA)) in CAMHS. This was part of the STADIA Trial (STAndardised DIagnostic Assessment for children and young people with emotional difficulties)—a randomised controlled trial assessing the effectiveness of the DAWBA in aiding clinician-made diagnosis decisions [10, 11].

The Development and Wellbeing Assessment (DAWBA) [12, 13] is an SDA tool designed to support decision-making around mental health diagnoses from the Diagnostic and Statistical Manual of Mental Disorders (5th Ed.; DSM-5) [14] and International Classification of Mental and Behavioural Disorders (ICD-10) [15] for children and adolescents. In the initial validation of the DAWBA, results were promising for its use as an epidemiological and clinical tool, with ‘substantial agreement’ between case note diagnoses and DAWBA results, though the DAWBA identified more comorbid disorders [16]. The DAWBA has been used for large nationwide surveys in the UK to assess prevalence of mental health disorders [17, 18]. A recent study in Denmark [19] comparing referrals to CAMHS with and without a DAWBA, found that the DAWBA group referral decisions were more sensitive and specific, with authors suggesting use of the DAWBA with referral letters may lead to more appropriate referrals to CAMHS. As demand for CAMHS exceeds capacity, the DAWBA has the potential to aid clinicians in making timely decisions about referrals and diagnoses which may help improve clinical capacity and service user experience.

The primary aim of this study is to identify the barriers and facilitators to the implementation and use of the DAWBA in CAMHS and to explore the contextual factors and causal mechanisms that might affect intended outcomes within the STADIA trial through exploring the experience of the DAWBA by different users and stakeholders.

## Method

### Design and frameworks

The study is a qualitative process evaluation nested within the STADIA trial of a standardised diagnostic assessment tool (DAWBA) in CAMHS (Trial registration number: ISRCTN15748675) [10, 11], and the full results of the trial have been reported elsewhere [11]. Qualitative data were collected through semi-structured interviews during two time periods: the internal pilot for the trial (2019–2020) and the main trial (2021–2022).

**Fig. 1:**
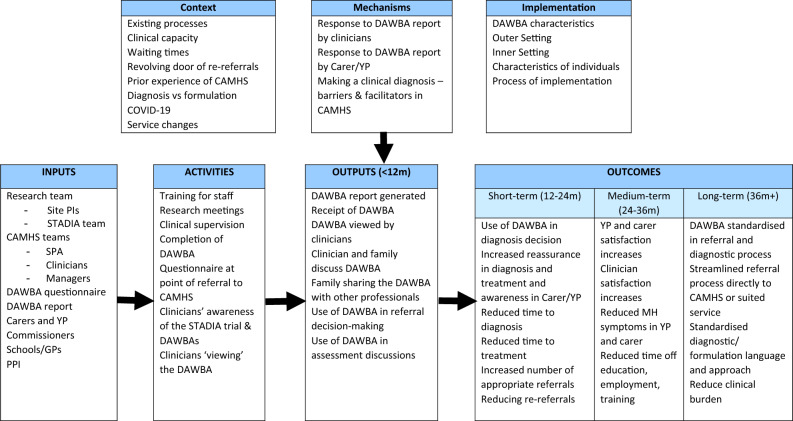
Initial Logic Model for STADIA Trial

Following the interviews during the internal pilot, an initial logic model was co-designed by the study team to further inform the interviews during the main trial (Fig. 1). This logic model represented the underlying theory of the DAWBA intervention evaluation in STADIA in a simple, diagrammatic form. The inputs and activities that were planned to occur as part of the trial were listed alongside the immediate expected outputs which were actions related to the generation and use of the DAWBA report within CAMHS. The short-, medium, and long-term outcomes included the primary and secondary outcomes from the main trial, as well as some interim steps (e.g. use of DAWBA in diagnosis decision) that weren’t formally measured. Contextual and implementation factors and key mechanisms that might influence the achievement of the expected outputs and outcomes were added based on the learning from the internal pilot. The purpose of developing this logic model was to aid understanding of the complexities of how the DAWBA may produce its intended outcomes and identify any underlying assumptions and risks. As such, this study adopted a critical realist approach [20], acknowledging that CAMHS represents a complex system within which the DAWBA report was intended to be used to benefit clinicians and patients but that other factors may influence its use. The critical realist approach combines ontological realism (acknowledging the existence of a reality independent of human perception) with epistemological relativism (recognizing that knowledge is socially and culturally mediated and constructed from a particular perspective). This separation of ontology and epistemology through critical realism may help to promote interdisciplinary research which aims to have a practical impact in relation to real-world problems [21]

Questions based on this logic model were added to the interview schedule in the second set of interviews (during the main trial) to collect more focussed data to test and develop the logic model. The Consolidated Framework for Implementation Research [22, 23] was used as the overarching framework for this study and guided the development of the logic model, the interview questions and the data analysis.

### Setting and intervention

STADIA took place in 8 CAMHS NHS sites across England [10], involving 1225 participants aged 5–17 years, with emotional difficulties, who had been referred to CAMHS (excluding emergency/urgent referrals which required an expedited assessment). Following randomisation, the DAWBA was completed by participants (parent and/or young person) in the intervention arm and a trial-specific DAWBA report (See Appendix 1 for the DAWBA Report Template) was prepared for each participant, which was shared with the CAMHS triage team (clinicians deciding about referral acceptance) and then retained within CAMHS clinical records for all clinicians to access. A copy of the report was also sent to the family. Whilst the DAWBA was the intervention, the primary outcome of the trial was clinician-made diagnosis decisions about the presence of an emotional disorder within 12 months of randomisation. Ethical approval was obtained from South Birmingham Research Ethics Committee (Ref. 19/WM/0133).

The study setting was affected by the context of the COVID pandemic. Sites participating in the study were required to move the clinical assessment process from face-to-face appointments to online video or telephone appointments due to lockdown restrictions. A full description of some of the benefits and challenges of moving appointments online during this study are described elsewhere [24]. In summary, there were some benefits in terms of reduced travel time, better accessibility and flexibility, which improved availability and engagement. However, there were other problems associated with remotely accessing mental health services, and some young people were not thought to engage as well with mental health professionals and clinicians also felt that online appointments lacked the provision of important body language cues which could reveal insights to help understand the patient more effectively.

### Participants

109 unique participants took part in semi-structured interviews across the internal pilot (n = 52) or the main trial (n = 57). Participants were recruited from all eight sites and reflected three groups: staff working in or alongside CAMHS (clinicians, service managers, service commissioners (funders) and embedded researchers); young people aged 16–17; and "parents/carers of children and young people referred to CAMHS.  Researchers working on the STADIA trial were included as they worked within the services and interacted directly with clinicians, managers and parents/carers and young people so provided a perspective on how the DAWBA was used and perceived by multiple stakeholders. Participant demographics and staff role breakdown are in Table 1.

**Table 1:** Participant demographics

	Young People	Parents/carers	Staff
Number interviewed	15	38	56
**Sites**			
Nottinghamshire	3	6	
Cambridgeshire	5	5	
London	4	6	
Pennine	0	10	
Berkshire	2	9	
Gloucestershire	0	2	
Doncaster	0	0	
Surrey	1	0	
**Gender**			
Male	2	6	–
Female	13	32	–
Other	0	0	–
**Ethnicity**			
White	11	34	–
Other ethnicity	4	4	–
**Age of index child**			
5–10	–	12	–
11–15	–	19	–
16–17	15	7	–
**Prior experience of CAMHS**			
Yes	4	12	–
No	7	23	–
Unknown	4	3	
**Staff role**			
Clinician	–	–	21
Team/Service Manager	–	–	14
Service Commissioner	–	–	6
Embedded STADIA Researcher	–	–	15

79% of young people and parents/carers interviewed were in the intervention arm and had completed the DAWBA. Of the staff interviewed, clinicians were all potential users of the DAWBA reports, whilst embedded researchers, managers and commissioners were stakeholders but not actual users.

To achieve maximum demographic variation for young people and parents/carers a purposive sampling strategy was used [25]. This enabled us to try to balance the sample across trial sites, to allow us to explore context specific variations and commonalities. We also purposively sought to recruit participants who were male and from ethnic minority groups who are often under-represented within mental health research. Staff demographics were not collected in order to preserve anonymity of participants. The principles of information power [26] were used to inform the final sample size. This goes beyond the concept of data saturation to consider the information power relative to the aims of the study, the specificity of the sample, the use of theory and the quality of the interview dialogue.

### Data collection

Semi-structured interviews were conducted by one researcher (KN), a female post-doctorate researcher with experience of interviewing young people and topics relating to health and health services. The interview questions were co-designed with the trial Patient and Public Involvement (PPI) lead (CE), and through consultation with the STADIA PPI Panels and clinicians in the trial management group and piloted with a small sample.

### Procedure

Participants from both arms who had consented to be contacted about the qualitative process evaluation study were sent further information. Of those who did not participate, most did not respond to the invitation in any way, but some identified that they did not want to participate due to them being in the control arm, having too much going on in their lives, having had a bad experience with CAMHS or not wanting to engage with anything CAMHS-related. For those who agreed to participate, an information sheet and consent form were provided. Following consent, interviews were arranged in-person, online via Microsoft Teams or by telephone, depending on participant preference. A £10 shopping voucher was offered to young people and parents/carers as a compensation for their time.

For staff, information about participating in the qualitative process evaluation study was communicated by the site Principal Investigators (PI). Email, team meetings and word-of-mouth were used to share details. Staff who were interested in participating were asked to contact the researcher (KN) directly. They were subsequently provided information sheets and consent forms.

All interviews were audio recorded via a Dictaphone and the encrypted audio data were fully transcribed prior to analysis. Interviews typically lasted between 30 and 60 min and notes were made by the interviewer to highlight key points. The transcripts were not checked by participants prior to analysis. The interviewer (KN) had no prior relationship with the participants, and she only shared her name, prior experience and purpose of the research with them.

### Analysis

Transcribed interview data were coded by KN and analysed in NVivo 12. The six steps of reflexive thematic analysis [27, 28] were followed which include: familiarisation by reading and re-reading transcripts; the coding of the transcripts; initial generation of themes from the codes; the review and further development of themes; refining and defining of themes; writing up the description of the themes. Open coding was used and these codes grouped together into categories and subsequently themes that were derived from the data. A framework approach was adopted [29] and the constructs and domains of the CFIR [22, 23] applied to provide the overarching framework for the themes, identifying how the themes identified in the data reflect specific aspects of the CFIR.

A selection of anonymised data extracts were also coded by the trial lead (KS), PPI lead (CE), a clinician (NT), and qualitative lead (LT) to check the coding and to ensure clinical, young people, and parents/carers perspectives were considered within the analysis. Further feedback on the themes and interpretation was provided by the trial management group including the site PIs, but not from the participants themselves.

### Reflexivity statement

During this work, we considered the axiology (our values and biases) and how this may influence our interpretation. The research team is multi-disciplinary, bringing multiple perspectives to the analysis and interpretation. The experience and background of the primary researcher (KN) and qualitative lead (LT) as psychologists, with relatively little prior experience of CAMHS, influenced our approach to data collection and interpretation of the data. As we are not clinical psychologists and do not have a history working in CAMHS this reduces some elements of bias and power dynamics with participants, We felt equally able to critically engage with the different narratives of both young people, parents/carers and CAMHS staff, but as psychologists we acknowledge assumptions and biases about psychological interventions which we may have brought to the study. The researchers’ positionality as critical realists meant that we were striving for rigour to understanding the reality of how the DAWBA was used and experienced, but also open to alternative interpretations, thereby offering a nuanced understanding of the interplay between agency, structure, and context. The contribution of the other authors to the analysis included the perspectives of clinicians (KS, AB, BD, TM, JG) and a parent with lived experience of seeking help from CAMHS for their child (CE). As the initial coders were not currently going through the CAMHS system (directly or as parent/carers), we cannot directly empathise with the experiences, which was why the PPIE lead and the PPIE groups were essential, and their feedback directly informed the paper.

## Results

Eleven themes were generated relating to factors influencing the experience and use of the DAWBA within the trial. Five of these acted as facilitators and six as barriers.

**Fig. 2:**
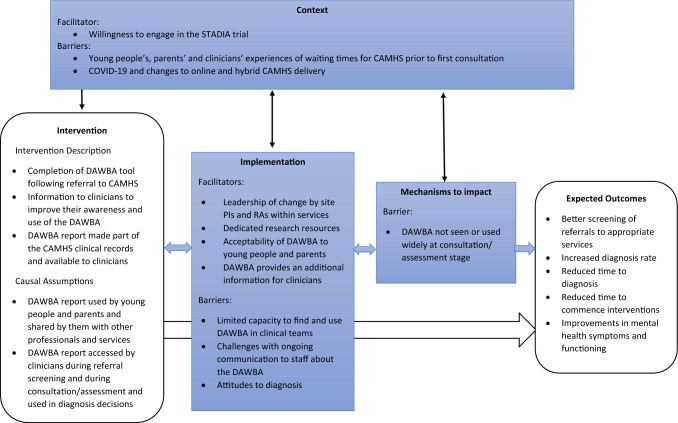
Summary of Process Evaluation Results (blue boxes), framed within STADIA Trial logic model (white boxes)

Figure 1 provides a summary of these facilitators and barriers identified through the analysis. It draws a distinction between those specifically tied to the process of *implementing* the DAWBA within the STADIA trial, those that were pre-existing *contextual* factors acting as broader facilitators and barriers influencing the delivery of the intervention, and those that related to the *mechanisms* that were expected to underpin the intended outcomes but were not sufficiently activated [30]. These core functions of the process evaluation (implementation, context and mechanisms) are represented in the blue boxes alongside the major components of the delivery of the intervention during the STADIA trial and the expected outcomes in the white boxes. Whilst not part of the process evaluation, these simplified elements of the trial logic model are vital for framing the process evaluation results [31] Fig. 2.

Table 2 maps the themes relating to barriers and facilitators to the use of the DAWBA identified in the analysis onto the structure of the CFIR domains and constructs [24]. These are described in more detail with illustrative quotes below.

**Table 2:** Participant demographics

Aspects of CFIR		Themes	
CFIR Domain	CFIR Construct	Facilitator	Barrier
Innovation	Innovation Design	Acceptability of DAWBA by young people and parents/cares	
	Innovation Design	DAWBA provides additional information for clinicians	
Outer Setting	Critical Incidents		COVID-19 pandemic and changes to online and hybrid services
Inner Setting	Work Infrastructure		Experience of waiting times for CAMHS prior to first consultation/ assessment
	Available Resources	Dedicated research resources	
	Available Resources		Limited capacity to find and use DAWBA in clinical teams
Individual Roles	Innovation Recipients	Willingness to engage in the STADIA trial	
	Implementation Leads	Leadership of change by site PIs and RAs	
Individual Characteristics	Capability		Challenges with ongoing communication with staff about the DAWBA
	Motivation		Attitudes to diagnosis
	Opportunity		DAWBA not seen or used widely at consultation/ assessment stage

### Facilitators

#### Willingness to engage in the STADIA trial

A number of internal motivators acted as incentives for young people and parents/carers to take part in the trial and demonstrated that it was feasible to recruit to the trial during the internal pilot. This contextual factor highlighted the role of ‘Innovation recipients’ (CFIR individual domain) being engaged with research and open to innovations to improve services.

Some reflected that engaging in this study gave them a sense of shared endeavour and identity that had some positive personal benefits for them. It made them feel less alone and was perceived as acknowledgement while waiting for an assessment, also providing additional information during that wait. A few also believed that participating in the study might increase the likelihood of their referral being accepted.

*“If I participate it is just the feeling that I feel less alone, with all this because sometimes it is very challenging and very tough” – Parent/carer (Control)*

Others were motivated to improve services through research and reflected on their negative prior experiences with CAMHS, including referral rejection and long waiting times. This inspired them to assist in seeking service improvements.

*“Research, yes I think it is quite good, because hopefully these Trusts will take that on board and try and make sure that people have better outcomes” – Parent/carer (Control)*

Some participants reflected on a sense of altruism, to contribute to the greater good and make things better for others.

*“that's the benefit, is that feeling that you are helping with something, that's a positive for me” Parent/carer (Intervention)*

#### Acceptability of DAWBA by young people and parents

The use of the DAWBA was met with predominantly positive perceptions by young people and parents/carers. They used the DAWBA report to help understand their symptoms or as a form of ‘evidence’ in seeking help from external services (e.g. schools, primary healthcare, private mental health services). This aspect of ‘Innovation Design’ (CFIR Innovation domain) promoted the active use of the DAWBA report by young people and parents.

Many participants were positive about the accessibility and presentation of the DAWBA report, which used colour coding and a graphical depiction to communicate the young person's emotional difficulties compared with population norms. The online access to completing the DAWBA was generally a facilitator, especially for young people.

*“The visualisation is good, it's really easy to understand the different levels that are there and then a really nice summary. I think that the format of the report is actually really, really well thought out as well.” – Parent/carer (Intervention)*

Parents and carers found the DAWBA report beneficial in providing clarity and points of reflection about the severity of their child’s symptoms. Putting their child’s symptoms in a population context offered reassurance to parents, particularly for those  children and young people whose DAWBA ratings showed them to be in the average range.

*it made you realise that there are people with bigger issues that what you think you may have...so it puts it into context what behaviour you are seeing may not be as severe – Parent/carer (Intervention)*

This reassurance was also reported by parents whose children had scored highly on the DAWBA, as they felt validated in seeking help. Parents also hoped that the added information would be useful in when seeking support from others.

*I have since been able to go to the GP and say look we have had the report through now, can we now actually look at getting [child] a diagnosis, [child] does have really severe anxiety, [child] does get depressive episodes and I don't think this just pretending it will go away is very helpful; … for us to have a report that shows these things and has validated [child]'s experiences and ours ... it has been received quite well, like a report gets listened more than we do.” – Parent/carer (Intervention)*

The report was also seen to have benefits for the child, both in terms of having something concrete for themselves, which acknowledged their experiences, and as an opportunity to open conversations between parents and the child.

*“[child] loved the report so, we got the report through, the first person I showed it to was [child] and then because I got it through on my phone I have been able to send it through to their phone and said to [child] look it is up to you who you share this with, this is your information, remember stuff you share with people cannot be taken back. I quite often find [child] sitting there having a read through of it” – Parent/carer (Intervention)*

#### DAWBA provides additional information for clinicians

Although few DAWBA reports were encountered by interviewed staff, they were considered as having the potential to provide additional information in clinical sessions. Clinicians could see benefits of ‘Innovation Design’ (CFIR Innovation domain) and their appraisal of the DAWBA was often in direct comparison to other tools used such as the Revised Child Anxiety and Depression Scale (RCADS) and Strengths and Difficulties Questionnaire (SDQ), generally viewing the DAWBA as “more thorough” with “more areas covered” and “more visual”.

*"I think the DAWBA is very thorough, ... the amount of detail that it goes into is helpful, I'm not a big fan of RCADS and SDQ ..." Clinician*

In Single Point of Access (SPA) teams, who make initial decisions about referral acceptance into CAMHS, there was better awareness of the DAWBA report and perceptions that it could help with referral decisions.

*“as part of our decision making about whether they come into CAMHS or not, we would review [the DAWBA report] and just have a look and see from that person's rating ..... so that's how we would use it to help us make decisions or maybe understand, okay yes you still have anxiety but that still means you don't need CAMHS but there might be another service that's more appropriate” - Clinician*

In subsequent clinical consultations, following referral acceptance, the DAWBA report was thought to be a useful starting point for conversation between clinicians, young people and their parents/carers, and to give further detail and expression regarding their experiences.

*“In terms of the emotion in the first appointment ... people are frantic to be reassured that you're not going to then just pass them on for another few weeks for another assessment and it felt that [the DAWBA report] had taken some of that out, it was something to focus on at the time” – Clinician*

Some clinicians described how it helped them prioritise which problems to focus on in an initial assessment.

*“the DAWBA helps me to think is it coming through more mood or anxiety or sleep problems or conduct problems, and then I'll focus the assessment” - Clinician*

Young people and parents perceived the report as providing additional information about the child that could be used by a range of healthcare professionals and education providers. They also saw the benefits of the report to clinicians if they were offered an assessment.

*“I think in terms of going for an assessment that it's going to be very helpful because it kind of has given a starting point to talk about and what doesn't need to be talked about. I think it will be very helpful and will cut short a lot of questions” – Young Person (Intervention)*

#### Dedicated research/administrative resources

Researchers working in each site invested the most time and labour in the study and assisted in the operationalisation of the DAWBA into clinical team practice. Having this embedded and dedicated team member was a key ‘Available Resource’ (CFIR Inner Setting domain). The critical tasks carried out to implement the DAWBA included supporting participant completion of the DAWBA, generating the DAWBA reports, sending them out, sharing and adding them to clinical records and highlighting its presence to clinicians. The clinicians commented that, without this support, incorporating the DAWBA into practice would need additional administrative resources.

*“a time element to the organisation, the administration, you know, all the elements of how it's sort of input into the system, how it's sort of, you know, managed, would need to have time” Clinician*

#### Leadership of change

Clinicians identified the importance of invested and enthusiastic leaders of change in facilitating the use of the DAWBA in services. PIs at each site were instrumental in this ‘Implementation Lead’ role (CFIR Individual Role domain) and pivotal in raising awareness of the DAWBA through emails and regular presentations to inform clinicians about the study. They and site researchers also engaged in ongoing active communication to increase clinician awareness and active searching for the DAWBA.

*“[The site PI] is a big advocate for [the DAWBA] and [the researchers] were very, very clear and always very keen to kind of talk and problem solve and support you with any questions you had around.” Clinician*

### Barriers

#### COVID-19 pandemic and changes to online and hybrid services

For the majority of interview participants, the COVID-19 pandemic was at the forefront of their experiences during the STADIA trial and therefore had a strong impact on how the DAWBA was used and on CAMHS generally. This broader contextual factor represented a ‘critical incident’ (CFIR Outer Setting domain) that had a dramatic effect on how CAMHS were delivered as well as on children and young people, parents and carers and CAMHS staff. New ways of working and of accessing mental health support meant changing from predominantly face-to-face appointments to online/virtual, and then changing again to a hybrid model, representing a significant change in practice, resources and service demand.

*“My main experience of coming across [the DAWBA], and this is part of the difficulty I think, because it came in during the pandemic, was everything became very disjointed, so in terms of being able to work around teams, you got your referrals, [but] didn't have that informal conversation that we're talking more about with your colleagues.” – Clinician*

The rapid changes in practice and procedure meant that the DAWBA was not at the forefront of clinician concerns. Communication was more disjointed than usual, making reminders about looking for the DAWBA reports in electronic records more difficult. This may have contributed to limited DAWBA reports being seen by the clinicians interviewed for this study.

### Experience of waiting times for CAMHS prior to first consultation/assessment

Long waiting times existed prior to COVID, but the pandemic exacerbated these and there was also a subsequent increase in referrals. This resulted in a considerable wait between referral and first consultation/assessment (if the referral was accepted). This contextual factor related to ‘work infrastructure’ (CFIR Inner Setting domain) and provided a backdrop to clinical interactions that were fraught with pressure, frustration and distress.

For many young people and parents/carers, the receipt of a DAWBA report during these extended waits provided some form of interaction and acknowledgement during the referral process. Receiving the report helped participants feel that they were getting something back, offering comfort in difficult times.

*“because I've not heard anything yet from the referral, it is quite nice to sort of have a bit of acknowledgement.” – Parent/carer (Intervention).*

However, clinicians felt under more pressure due to the waiting times and increases in clinical severity resulting from extended waits. They described how these demands on services led to a tendency to focus on existing practice and tried-and-tested approaches rather than being open to innovations and new methods.

*“if you've high waiting lists and high levels of people complaining of how long they're waiting for the service and people getting worse because they've been waiting that long for this service then the priorities will shift to how do we offer treatments ... so if you're in survival mode as a service then innovation becomes a secondary thing because we might not be here next to implement this new innovation if we don't survive this current storm that we're in” - Clinician*

### Limited capacity to find and use DAWBA in clinical teams

Clinicians were concerned about the additional workload and time needed to be aware of and engage with the DAWBA report: to find it in a patient’s electronic clinical records, read it, and use it within the clinical assessment.

*“[you] need to have time, and I think time is something that none of us have much of, so it would be how and who and where that time is going to come from.” Clinician*

The context of high pressure on clinicians meant that, in practical terms, clinicians felt that they had limited capacity to incorporate additional information to existing processes. A specific challenge was related to the time needed to find the DAWBA report within a patient’s electronic clinical records, which, in the context of high demands and time pressures, was seen as a considerable barrier to using it. Clinicians reported electronic clinical records as generally “very cumbersome” and making it “really challenging” to find things, which made introducing new tools or documents more difficult.

*“[The electronic clinical records] would be a big barrier to using the DAWBA .... it's just all the pressures of work that would get in the way of finding the time to do it” Clinician*

Combined with the time needed to get clinicians to change their routine practices, which was difficult when they felt under pressure, this meant that adapting usual practice to find and use the additional information within the DAWBA report was met with considerable indifference.

*“where would you find the time to retrain, get people to start thinking a different way; and then you think of the priorities that you currently have” Clinician*

### Challenges with ongoing communication to staff about the DAWBA

Given the context of long waiting lists, pressure on staff time, and the tendency to revert to usual practice, staff reported needing more frequent communication and convincing information about the DAWBA to keep it at the forefront of their minds and encourage its use. Without regular reminders to check for the DAWBA report in electronic clinical records or ask patients about it, a barrier to use arose from the lack of competence and knowledge reflecting the ‘Capability’ (CFIR Individual Characteristics domain) of clinical teams. The form of communication was highlighted as being important and, despite concerns about time pressures, clinicians still preferred direct, in-person meetings about the DAWBA and the trial. These were perceived to be more effective than emails, even though they were more challenging to arrange. Most clinicians were unaware of emails or newsletters sent from the STADIA team due to the volume of emails clinicians receive and the tendency for these to go unnoticed. It was felt that in-person meetings and presentations by persuasive and trusted people were a better form of information.

*“I think if it's to do with people, you know, I think that's always the clincher, if it just comes in an email form or it's in some kind of newsletter, nobody's going to see it or notice it, so yes, how creative people can be and meeting with individuals, meeting with teams” Clinician*

There was also some confusion about the validity of the DAWBA tool and whether it was an established measure or one that was being tested. Some participants identified a need for more engagement with clinicians and information on the DAWBA at the start of the trial, specifically raising awareness to a wider range of clinicians who could come across it. This would include what the DAWBA report looked like and where it could be accessed (although, throughout the trial, this was done regularly through site PIs and researchers).

*“[We needed to] introduce and give more information about the DAWBA because I think they'd never heard of it and they didn't know enough about that it was standardized tool and that it had good reliability and validity, it was just something that I think they worried about” Researcher*

### Attitudes to diagnosis

There was some reluctance to use the DAWBA stemming from individuals’ general attitudes to diagnosis. This clearly acted as a barrier to using the DAWBA which was related to individuals’ commitment or ‘Motivation’ (CFIR Individual Characteristics domain). Many clinicians described a preference for a formulation approach over diagnosis as this was considered more holistic, less stigmatising and avoided labelling in a population where presentation of symptoms may change with age.

*“100% [prefer formulation], as putting kids into nice, neat diagnostic boxes just feels unrealistic, or for anybody.” Clinician*

The DAWBA, with its diagnosis-based language and focus, is clearly aimed at facilitating the diagnostic process, bringing into question the suitability of the tool in services that prefer formulation. There was also a sense of reluctance to engage with diagnosis and diagnostic tools from staff who were not from medical backgrounds with the general belief that only medical doctors made diagnoses.

*“some are quite anti-diagnosis if you like. There is a very mixed consensus isn't there, around how we use diagnosis, whether we should, how useful the language is, etc and of course the DAWBA report itself is based off the DSM isn't it and the ICD 10 I think?” Researcher*

However, there a variety of views on the role of a diagnosis were expressed. Young people and parents/carers generally expressed desire for a diagnosis in order to access support from other services.

*“If they diagnose you, they actually have to help you.” Young Person*

Other professionals were concerned that a diagnosis was essential for some interventions (e.g. educational, medication) and a reluctance to diagnose may impact young people who need support. Commissioners also articulated the importance of data on diagnosis for funding of services.

*“diagnosis can sometimes open a door for accessing other services and funding as well as being containing, reassuring, so yeah it can definitely be really helpful.” Clinician*

### DAWBA not seen or used widely at consultation/assessment stage

It became evident that DAWBA reports were not necessarily being used in the way expected or articulated in the logic model. The main hypothesised mechanisms through which the DAWBA would affect outcomes was by clinicians accessing and using the DAWBA reports at two stages: (1) triage—as part of the decision to accept or reject a referral; (2) assessment—for accepted referrals, during initial assessment consultations to help inform diagnostic decisions. However, few clinicians interviewed had seen or used a DAWBA report, particularly in stage 2. This could be partly due to proportion of referrals that were rejected but also challenges finding the report in the electronic clinical records. One clinician identified the need for a DAWBA report to be flagged so it was more obvious to clinicians.

*“I guess one thing that would be helpful is if when you saw the client and a DAWBA had been completed there was an alert that comes up on [the electronic clinical record] just saying this client has a DAWBA ... and then a link to where to go and check it so that you're not having to trawl through notes to see that, so that would be helpful because I didn't see that until after that choice appointment.” Clinician*

For those that had seen a DAWBA report in the electronic clinical records, there was a reluctance to use it if a long period had passed between DAWBA completion and the actual clinical consultation. This seems to reflect a lack of ‘Opportunity’ (CFIR: Individual Characteristics Domain) for clinicians to use the DAWBA reports in the way intended during assessment consultations. Furthermore, some DAWBA reports had discrepancies with the clinical formulation or diagnosis. Sometimes this was discussed with the patient but some clinicians chose to ignore the DAWBA when this occurred.

*“the clinical interview did not marry up very well with what the DAWBA kind of revealed so I guess it made me more aware that certain areas may need further exploration... With children and adolescents in particular, things change over time and are transient so maybe when the DAWBA was completed that was entirely accurate but actually things may have progressed in a different way” - Clinician*

However, many did see a potential benefit of using the DAWBA to inform the referral process, as a pre-referral screening tool. This could bring benefits to both child or young person and the service by informing which referrals were most appropriate for CAMHS intervention and which better signposted elsewhere. Commissioners and clinicians were also positive about the potential of the DAWBA for engagement during long waiting times, informing referral acceptance or redirection to other services, or in initial assessments. This ‘Adaptation’ (CFIR: Implementation Process) happened during the implementation of the DAWBA into sites.

*"I'd be keen to think about how that may be complemented in [the single point of access] service." Manager*

This would potentially reduce clinical burden by taking place before assessment (as situated in the trial). Others felt that to be most beneficial clinically, the DAWBA should be completed closer to assessment so the information was more up to date.

*“it's quite a detailed assessment of that young person and from a referral screening perspective, those that had participated in [completing] the DAWBA, it was really really useful in terms of offering them their initial assessment because you have so much more additional information that was actually coming from the young person and the family as opposed to what a GP might write, or a school might write in a referral, which is only their perception, so it's really helpful, […] It's been used as a way of thinking about the referral we've had in and making a more informed process of decision about what's going to be right support for that young person. And then I guess if they come into the CAMHS treatment pathway then it informs that clinician who's picking them up about where they were at, so although it may be a long waiting list there's still an element you can then match, you can then sort of gauge how they've progressed and what's you know got better, what's not. You've got a baseline really to look at.” – Manager*

## Discussion

This paper describes the process evaluation for the STADIA trial in which the DAWBA was tested within CAMHS in 8 NHS sites to support clinician-made diagnostic decisions [10]. We identified several barriers and facilitators to the use of the DAWBA in the trial which related to contextual factors, aspects of how the DAWBA report was used in practice and causal mechanisms that appear to have affected the intended outcomes from the trial. Insights into the unique challenges of using new diagnostic tools and processes in over-stretched, highly pressured CAMHS were gained which can provide future guidance to service-related, research and implementation activities.

For young people and parents/carers, there were high levels of engagement with the DAWBA and an overall willingness to engage in the trial. There was a sense of reassurance and acknowledgement in receiving the DAWBA report during long waiting times, and it was thought to provide tangible ‘evidence’ that provided validation of symptoms. For young people and their parents/carers, this suggests that the DAWBA report could act as an enhancing mediating factor – improving their experience during the long waiting time whilst not affecting efficiency or clinical outcomes specifically [7]. The DAWBA report was also perceived by young people and parents/carers to have the potential to improve access to other services with the information it provided, actively being used in the absence of any other validated information from health professionals. However, outcome data from the STADIA trial found no effect on clinical outcomes [32]. Furthermore, further research is needed to understand how a DAWBA report is used and interpreted by other services and professionals, such as GPs and schools.

The barriers to using the DAWBA during the trial related to lower levels of engagement from clinical teams. This was due to a range of contributing factors which highlight important processes involved in bringing about changes to routine practice which weren’t in place for this research trial. Staff felt that they had limited capacity to adopt a change to their usual practice due to the highly pressured context of services, further compounded by the impact of the COVID-19 pandemic on referral rates and workloads. Whilst referral numbers initially reduced at the start of the pandemic, they then increased sharply. The already limited staff resources became even tighter with staff burnout and sickness also a key issue [33]. Some children and young people were easier to contact for the study due to isolation and the online nature of the DAWBA, however their mental health was often significantly worse [34]. For staff working in pressurised CAMHS with long waiting lists of increasingly desperate patients with worsening mental health, there appeared to be insufficient time and capacity to read communications about the study, to understand the DAWBA report. This was compounded by practical challenges of finding the DAWBA report in electronic clinical records to enable it to be used in the intended way. More frequent communication with frontline clinical staff may have improved clinician understanding and confidence in using the DAWBA reports. Clinical engagement and capacity in other CAMHS-based studies has been increased with fortnightly meetings to encourage ownership and responsibility for the study within the team [9] and collaboration with community settings as a way to manage limited resources [35]. However, time pressures within packed and busy clinical team meetings, with full agendas, meant that this frequency was not feasible for STADIA. Furthermore, as a stand-alone RCT, any changes in clinician practice to use the DAWBA reports were not consistent – clinicians might see a few patients with a DAWBA in their electronic records, but many others without one. The additional cognitive load, alongside significant existing demands, required to remember to look for a DAWBA report that may only be infrequently present seems to have been a notable barrier to this key mechanism.

There were general concerns about the validity and value of diagnosis and many clinicians voiced a reluctance to use diagnostic labels and language associated with ‘disorders’ due to their stigmatising potential. Clinicians’ attitudes towards the use of SDA tools and outcome monitoring have been identified in previous studies as a potential barrier to their use in CAMHS [36, 37]. These studies suggested that staff see a use for SDAs as an adjunct to, but not a replacement for, clinical assessment. In STADIA, the DAWBA was also introduced as an adjunct within real-world services. However, disinclination to make diagnoses and limited understanding and trust of the DAWBA as an SDA tool were at the forefront of clinicians’ reluctance to use it. Concerns about the length of time between DAWBA report generation (at referral) and initial assessment (often many months later) also contributed to a lack of trust in the DAWBA report. This highlights the importance of key attributes such as the perceived advantages, credibility and risk of the DAWBA which influenced its use as a new innovation [4].

We also identified a number of facilitators which eased the use of the DAWBA in CAMHS during the trial. The provision of clinical leadership and research-based resources were critical. Through championing the DAWBA, administering it, adding it to clinical records and providing repeated communication to clinicians, the researchers provided important support rather than further straining clinical capacity. This highlights the importance of appropriate administrative resources for implementation which has been echoed in other CAMHS process evaluations [8]. However, despite this, the DAWBA was not integrated into practice as anticipated due to a variety of internal factors as discussed above (capacity and time restraints, difficulties accessing the report in electronic clinical records, clinician views on diagnosis etc.) and external factors (COVID and long waiting lists). Whilst few clinicians interviewed had used the DAWBA report, there was acknowledgement that the report was informative and easy-to-use and could provide benefits as an initial focus for a discussion, which could often be quite stressful and fraught due to long waiting periods before that first appointment. However, more staff saw the benefits for the use of the DAWBA as an aid to screening referrals by SPA teams at the time of its completion in order to reduce waiting times and identify appropriate care pathways. This shows how the DAWBA could act a catalysing mediating factor—speeding up and/or improving efficiency of an intervention or service [7]—and potentially offer a reprieve during long waiting times for an assessment, highlighted as an issue for children, young people and staff.

Together, these barriers effectively meant that one of the main mechanisms through which the DAWBA report was intended to achieve its outcomes (active use in referral acceptance and clinical decision-making) was not fully activated during the STADIA trial. Assessing these factors through the lens of organisational change theories provides further insights into the nature of these barriers and how they might be overcome in future research and practice. There were insufficient triggers activated to support the level of engagement and buy-in to the changes in clinical practice required for the trial which meant that the social equilibrium within CAMHS was not disrupted sufficiently to activate the key mechanism of the DAWBA being seen or used at the assessment stage. Using Lewin’s Force Field Model of organisational change [38, 39] would suggest that there were not enough assisting forces (recognition of the need to change, top level commitment to embedding a change) to be able to overcome the resisting forces (preferences for existing ways of doing things, uncertainty about the validity of the proposed change and insufficient time/resources to change practice) which would result in current behaviours being maintained. Additional levers to encourage change can be identified which highlight the socially mediated nature of the diffusions of innovations in healthcare [4, 40], importance of social networks, opinion leaders, champions and boundary spanners [4]. These are important roles in organisational change and behaviour change which support the identification and adoption of new innovations and development of new social norms and behaviours to bring about change. Behaviour change interventions which include a social norm element (i.e. information on how peers behave) can help bring about change in the clinical behaviour of health workers [41].

The barriers also highlight the challenges of bringing about temporary changes to usual practice for the purposes of research. This was not an implementation study in which the DAWBA was to be adopted as a long-term change to practice which included significant effort to bring about a service-level change in how everyone was working. Rather, it was a real-world effectiveness trial requiring the short-term implementation of a new assessment just for the duration of the research. The study highlights some of the challenges of implementing complex changes for the purposes of an individual randomised effectiveness trials, and future research could consider the potential benefits of a cluster RCT which may be able to support changes to practice more effectively.

### Strengths and limitations

This study benefitted from a large sample size (n = 109) of multiple users and stakeholders across 8 different CAMHS in the UK. This allowed us to explore the variety of experiences and perspectives on the DAWBA and how it was used in practice. With two phases of interviews spanning different periods of time across the COVID-19 pandemic, we were also able to describe some of the impacts of the pandemic on the experiences of young people, their parents and carers and clinical and other NHS staff as well as the adaptations made to working practices in CAMHS.

However, the trial itself was also impacted by the COVID restrictions imposed, which came into force early in the project. NHS staff were under enormous pressure to adapt their working practices, and referrals were rapidly changing in volume and nature of presentations with referral numbers and complexity increasing [24, 33, 34]. Therefore, NHS staff may not have had as much capacity to engage with the study as they would have pre-pandemic.

The DAWBA was tested with a restricted range of children and young people referred to CAMHS, reflecting trial inclusion criteria—only those whose referrals were considered ‘routine’ and not ‘high risk’ were invited to take part in the trial. Many referrals were not accepted into CAMHS and therefore few DAWBA reports were seen by clinicians at assessment stage. While testing this referred population allowed the tool to be assessed pragmatically, it does not give insight into the use of the DAWBA in patients whose referral was already accepted, which may in turn have increased the frequency of DAWBAs seen. Future research may wish to consider testing and implementation of the DAWBA as standard for all levels of risk, with appropriate safeguarding for referrals scoring very high.

A further limitation is the relatively narrow scope of children and young people included in the interview study. As only young people aged 16–17 years were interviewed as part of this study, future research may wish to explore the specific experiences of under-16 year-olds to effectively implement into CAMHS. Furthermore, in the main STADIA trial, one-fifth of participants in the intervention arm didn't complete the DAWBA. Future research should also explore the barriers for this specific group.

### Recommendations for practice

The results of this study focus on the use of the DAWBA as part of an RCT, rather than a full-scale implementation and permanent change to clinical practice. As such, this research may have modest generalisability outside of research so offers recommendations in relation to this context.

There are benefits to engaging with children, young people and their families/carers soon after a referral is received by CAMHS. Whilst waiting for referrals to be processed, they valued being asked to provide information and for the results of this to be shared back with them.

Given the size and complexity of CAMHS within NHS Trusts, it was not feasible or within the scope of the trial to engage in a large change project to bring about consistent changes to practice. However, the use of the DAWBA as a screening tool by clinical teams at the ‘front door’ offers useful additional information which appears to be more acceptable to use than later in the pathway. Where additional assessment information is provided to clinicians there needs to be consideration about how accessible this is in electronic records and how it might be flagged to save time.

## Conclusion

This qualitative process evaluation of the effectiveness of the DAWBA in CAMHS commenced just before the COVID-19 pandemic. As such, changes to practice in how clinicians used the DAWBA had to be adapted to fit the rapidly changing context of lockdowns and virtual support. However, its use in clinical practice in assessments, following referral acceptance, was limited and impacted by limited capacity, difficulty finding the report in clinical records and a general reluctance towards diagnosis. Clinicians could see the benefit of increased information provided in an accessible report that could help with referral decisions at the ‘front door’ of services as well as facilitating conversations with young people and families. There was a positive response towards the DAWBA from young people and parents/carers, who reported improved understanding of their symptoms and sharing the DAWBA report with other services as evidence of their difficulties.

## Supplementary Information

Below is the link to the electronic supplementary material.

Supplementary file1 (DOCX 113 KB)

## Data Availability

No datasets were generated or analysed during the current study.
